# Beat-induced fluctuations in auditory cortical beta-band activity: using EEG to measure age-related changes

**DOI:** 10.3389/fpsyg.2014.00742

**Published:** 2014-07-11

**Authors:** Laura K. Cirelli, Dan Bosnyak, Fiona C. Manning, Christina Spinelli, Céline Marie, Takako Fujioka, Ayda Ghahremani, Laurel J. Trainor

**Affiliations:** ^1^Department of Psychology, Neuroscience & Behaviour, McMaster UniversityHamilton, ON, Canada; ^2^McMaster Institute for Music and the Mind, McMaster UniversityHamilton, ON, Canada; ^3^Centre for Computer Research in Music and Acoustics, Stanford UniversityStanford, CA, USA; ^4^Rotman Research Institute, Baycrest HospitalToronto, ON, Canada

**Keywords:** neural oscillation, time perception, electroencephalography (EEG), musical rhythm, child development

## Abstract

People readily extract regularity in rhythmic auditory patterns, enabling prediction of the onset of the next beat. Recent magnetoencephalography (MEG) research suggests that such prediction is reflected by the entrainment of oscillatory networks in the brain to the tempo of the sequence. In particular, induced beta-band oscillatory activity from auditory cortex decreases after each beat onset and rebounds prior to the onset of the next beat across tempi in a predictive manner. The objective of the present study was to examine the development of such oscillatory activity by comparing electroencephalography (EEG) measures of beta-band fluctuations in 7-year-old children to adults. EEG was recorded while participants listened passively to isochronous tone sequences at three tempi (390, 585, and 780 ms for onset-to-onset interval). In adults, induced power in the high beta-band (20–25 Hz) decreased after each tone onset and rebounded prior to the onset of the next tone across tempo conditions, consistent with MEG findings. In children, a similar pattern was measured in the two slower tempo conditions, but was weaker in the fastest condition. The results indicate that the beta-band timing network works similarly in children, although there are age-related changes in consistency and the tempo range over which it operates.

## INTRODUCTION

Humans often spontaneously match the timing of their movements to the timing of a predictable external stimulus, such as that found in most music (Drake et al., 2000b; see Repp and Su, 2013 for a review). Despite the ease with which people entrain to auditory beats, it is a complex process. First, the underlying beat must be extracted from the auditory stimulus, then communicated to the motor system, and finally the motor output is made in a predictive fashion, time-locked to the ongoing beat (see Zatorre et al., 2007; Repp and Su, 2013 for reviews). Rhythm perception and production are thought to share a common timing mechanism (Ivry and Hazeltine, 1995; Ivry, 1996; Hommel et al., 2001; Kello, 2003; Repp et al., 2011; Patel and Iversen, 2014) and there is evidence for a bidirectional relationship between these processes. For example, studies using functional magnetic resonance imaging (fMRI), positron emission tomography (PET) imaging, and magnetoencephalography (MEG) clearly show that listening to predictable temporal patterns recruits both auditory and motor regions of the brain in adults (e.g., Penhune et al., 1998; Schubotz et al., 2000; Schubotz and von Cramon, 2001; Grahn and Brett, 2007; Chen et al., 2008; Bengtsson et al., 2009; Fujioka et al., 2012). Conversely, motor activation influences the perception of auditory information (Phillips-Silver and Trainor, 2007; Su and Pöppel, 2012; Manning and Schutz, 2013). There is even evidence that the way in which infants are bounced to a rhythmic pattern affects their auditory interpretation of that pattern (Phillips-Silver and Trainor, 2005). At the same time, the ability to intentionally entrain movements to an auditory beat in tapping tasks or tasks involving other body movements takes time to mature (e.g., Drake et al., 2000a; Eerola et al., 2006). This raises the question of whether this extended immaturity of rhythmic entrainment reflects an immature ability to perceive regular beats in auditory cortex, or whether it is due to motoric immaturity. In the present study, we begin to address this question by examining whether auditory cortical responses to regular beats are mature in 7-year-old children. Specifically, we use electroencephalography (EEG) to examine auditory cortical entrainment to isochronous sequences at different tempi, bypassing the need for children to make an overt motor response.

The brain encodes different aspects of timing information, processing both *interval* (duration) and *relative* (beat-based) timing (e.g., Koch et al., 2009; Grube et al., 2010; Teki et al., 2011). Although young infants process both interval and relative timing, as discussed below, even the most basic aspects of time perception continue to improve for many years. For example, with respect to interval timing, young infants can detect small gaps in auditory stimuli (Werner et al., 1992; Trehub et al., 1995; Trainor et al., 2001; Smith et al., 2006) and detect duration differences in speech (e.g., Eilers et al., 1984), but thresholds do not reach adult levels until at least into the school age years, particularly when timing is compared across stimuli with different frequencies (Trehub et al., 1995).

There is evidence that even young infants are sensitive to relative (beat-based) timing when there is no requirement for motor entrainment. For example, they recognize rhythms across changes in tempo and frequency range (Chang and Trehub, 1977; Demany et al., 1977; Trehub and Thorpe, 1989) and extract the underlying regular beat (Hannon and Trehub, 2005a,b; Winkler et al., 2009). Older children can detect changes in underlying regularities in rhythmic stimuli (e.g., Baruch et al., 2004) and determine whether an isochronous beat train superimposed on a musical excerpt is at the correct tempo and phase (Einarson and Trainor, 2013). However, the ability to entrain movements to rhythm patterns or regular predictable beats remains immature for a long time. Infants as young as 5 months of age will move their bodies rhythmically and with more vigor when listening to music or isochronous tones as opposed to non-musical speech, however, these movements are not synchronous with the beat of the music (Zentner and Eerola, 2010). Two- and four-year-old children spontaneously sway and hop to musical stimuli, but do not adjust the tempo of this movement in response to fluctuations in stimulus timing (Eerola et al., 2006). Synchronization abilities do improve throughout childhood, but even 10-year-olds do not tap to an auditory rhythm as consistently as do adults (Drake et al., 2000a). In addition, even as these synchronization abilities improve, children’s spontaneous tapping rates remain faster than those of adults (Drake et al., 2000a; Baruch et al., 2004; McAuley et al., 2006). Together these studies suggest that although young children are inclined to move in a rhythmic fashion in the presence of a predictable auditory beat, they are unable to consistently entrain their movements to the underlying pulse. It is unclear if this is because auditory cortical entrainment mechanisms are mature in children and the immaturity lies in connections between auditory and motor areas, or whether the perception of entrainment in auditory cortex itself has a protracted developmental trajectory. The present study begins to address this question by focusing on the maturity of auditory cortical activity during rhythm perception in children.

One popular theoretical framework for understanding auditory rhythm perception is Dynamic Attending Theory (Jones and Boltz, 1989; Large and Jones, 1999). This theory proposes that internal oscillators entrain to external auditory rhythms in a way that focuses attentional resources at important points in time. For example, in an isochronous sequence, attention is focused toward the predicted onset time of the next tone. Neurophysiological evidence suggests that the underlying mechanism for predictive timing is reflected in oscillatory neural activity (Snyder and Large, 2005; Lakatos et al., 2008; Fujioka et al., 2009, 2012; Iversen et al., 2009; Arnal, 2012; Arnal and Giraud, 2012; Nozaradan et al., 2012). Neural oscillations involved in predictive timing appear to span the delta (0.5–3 Hz), beta (15–30 Hz), and gamma (30–100 Hz) ranges. Human beat processing is optimal when the tempo of the stimulus is in the range of 1–3 Hz (e.g., Fraisse, 1982; Drake and Botte, 1993; Large, 2008) and stimuli consisting of rhythmic patterns give rise to oscillations in the EEG in this delta range most likely because it directly reflects the frequency of the beat or its metric organization as phase-locked neural activities (Nozaradan et al., 2012). These oscillations reflect to some extent stimulus event onsets, but instructions to imagine different metric structures (such as accents every second beat vs. every third beat) while listening to the same stimulus modifies oscillation amplitudes, indicating that the oscillations can also reflect internally generated timing expectations (Nozaradan et al., 2011). Such modulation of oscillatory neural activities also contributes to the phase-locked neural activities across a wide range of brain areas related to auditory and motor systems (Fujioka et al., 2010).

Oscillations in the beta-band appear to be particularly important for auditory rhythmic entrainment (Fujioka et al., 2009, 2012; Iversen et al., 2009; Patel and Iversen, 2014), which is interesting given that the beta-band is also implicated in regulating the initiation of movement (e.g., Hari and Salmelin, 1997; Pfurtscheller and Lopes da Silva, 1999; Gilbertson et al., 2005). Furthermore, there is coupling between different oscillation frequencies, such that the amplitude of beta oscillations can vary according to the phase of a delta rhythm (Lakatos et al., 2008; Fujioka et al., 2012). While delta oscillations tend to phase lock to onsets of predictable sound events, beta oscillations typically do not. Thus, averaging evoked MEG or EEG responses to the sound events in a presented rhythm pattern may remove most activation in the beta-band. However, if single-trial analysis is used, fluctuations in beta-band power are evident (induced beta oscillations) that follow the beat of the rhythm pattern. Fujioka et al. (2009, 2012) measured induced beta responses to isochronous sound streams presented at three stimulus rates (tempi). They found beta desynchronization after each tone followed by a rebound whose timing depended on stimulus rate, suggesting that the rebound rate was predictive of the expected onset time of the next tone. The authors interpreted these results as being consistent with literature suggesting that beta desynchronization reflects a change in event processing (Pfurtscheller and Lopes da Silva, 1999), while synchronized rebound is associated with a “reset of an existing cortical network” (Pfurtscheller et al., 2005). Furthermore, this modulation of induced beta activity was found not only in auditory cortex, but also in a number of cortical and subcortical areas of the motor network. As participants were instructed not to move throughout the experiment, this suggests that even the perception of auditory rhythms may involve processing loops between auditory and motor regions.

Induced oscillatory neural activity representing beat perception has not previously been measured in children. In the present study we compared induced beta oscillatory activity from auditory cortex in 7.5-year-old children and adults while they passively listened to isochronous tones at three tempi. We expected that induced beta oscillations would be qualitatively similar across the age groups, but that differences might exist in the extreme (fastest or slowest) tempo conditions. The present study used EEG to examine beta-band activity, as opposed to MEG as in previous studies (Fujioka et al., 2009, 2012). Although spatial resolution is better in MEG compared to EEG, it is much easier to use EEG with children, as the demands for remaining completely still are less critical (Trainor, 2012). Importantly, the use of EEG will allow direct comparison to future studies in younger children and infants.

## MATERIALS AND METHODS

### PARTICIPANTS

Seventeen normal hearing adults (three males; mean age = 21.1 years ± 3.1; one left handed) and 16 normal hearing children (eight males; mean age = 7.6 years ± 0.2; one left handed, one ambidextrous) participated in the experiment. Two additional adults and one child were tested, but were excluded due to equipment failure. One additional adult was tested but was excluded due to reported hearing loss.

After the nature of the study was described, each participant (and for the children, also the parent) provided informed consent and completed a short survey outlining the participant’s hearing and musical history. None of the participants reported any family history of hearing impairment. Musical training was reported for eight of the children (*M* = 2.1 ± 1.9 years of formal instruction for the eight children) and all of the adult subjects (*M* = 8.9 ± 4.3 years of formal instruction).

The children were recruited through the Developmental Studies Database at McMaster University. The procedures used in this study were approved by the McMaster Research Ethics Board.

### EEG RECORDINGS

Each participant was seated comfortably in a sound-attenuating room. 128 electrodes were applied in a cap and their locations digitized (Polhemus Fastrak, Colchester, VT, USA). EEG was continuously recorded at 2048 Hz filtered DC to 200Hz using a Biosemi ActiveTwo amplifier (Cortech Solutions, Wilmington, NC, USA) with an active reference system. Before recording, electrode offset voltage was verified to ensure adequately low electrode impedance.

### PROCEDURE

The tone used for the stimulus sequence was identical to that used by Fujioka et al. (2012), and consisted of a 264 Hz pure tones with 8 ms rise and fall times and 45 ms steady-state duration. The tones were presented in isochronous sequences at the three tempi used by Fujioka et al. (2012), with onset-to-onset intervals of 390, 585, and 780 ms. Each participant experienced all three tempo conditions in each of the two blocks of the experiment, with the order of the tempo condition presentation for each block randomized across participants. In each block, ~350 tones were presented per tempo, for a total of ~700 tones per tempo condition across both blocks. These tones were sequenced using the program Presentation (Neurobehavioral Systems, Berkeley, CA, USA), and presented binaurally to participants through ear inserts (Etymotic ER2) at an intensity of ∼60 dB SPL. Participants passively listened to the stimuli while watching a silent movie with subtitles and were asked to minimize movements and eye blinks during testing phases. Once EEG recording began, the entire experiment lasted for about 20 min.

## DATA ANALYSIS

### AUDITORY SOURCE LOCALIZATION

**FIGURE 1:**
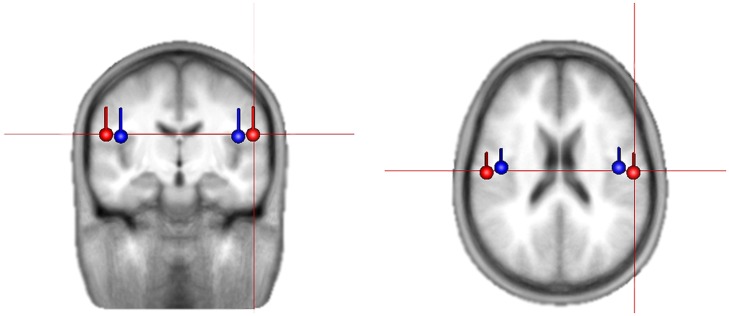
**Average location and orientation of the equivalent current dipole sources fitted in the bilateral auditory cortical areas for children (in red) and adults (in blue) that are used as spatial filter in extracting source activities**.

Using brain electrical source analysis (BESA, Gräfelfing, Germany) software, for each person, the averaged event-related potential (ERP) to the tones in the 780 ms condition was calculated (the slowest tempo gives the best signal-to-noise ratio in the evoked response; e.g., Näätänen and Picton, 1987). To do this, epochs were extracted from 100 ms before tone onset to 250 ms after tone onset, and epochs with high amplitude voltage were rejected such that 80% remained (e.g., see Gander et al., 2010 for use of percentage rejection). The epochs remaining after artifact rejection were averaged, and the resulting evoked response was filtered 1–20 Hz. The baseline was corrected using a 100 ms window prior to the stimulus onset. In order to examine beta activity from auditory cortex, the first step is to locate this region. For this, the N1 response was used, as it is a robust response known to be generated primarily in auditory cortex. The N1 response was identified as the most negative peak between 90 and 120 ms from tone onset in the global field power. Two symmetrical regional sources were fit, in each individual, to a window ± 10 ms around the identified peak and then converted into independently oriented single sources. The right source position and orientation was used as a spatial filter in the subsequent analyses. All participants’ final source locations were consistent with expected locations in the auditory cortex (see **Figure 1** for averaged dipole locations).

### INDUCED RESPONSE

Raw data were filtered using each subject’s right auditory cortex source as a spatial filter, and further analyses were performed on this source waveform. These data were epoched around each tone according to tempo condition (390 condition: -200 to 600 ms; 585 condition: -300 to 900 ms; 780 condition: -400 to 1200 ms). All individual epochs were down-sampled at 1000 Hz, filtered from 3 to 200 Hz, and exported for further processing in MATLAB.

Artifact rejection was performed in MATLAB by eliminating epochs with high amplitude voltage such that 80% of the epochs remained in each condition, and further analyses were done on these epochs. For each tempo condition, the waveforms remaining after artifact rejection were averaged to obtain an estimate of the evoked response, and this was subtracted from each individual epoch to obtain an estimate of the non-phase-locked induced response on each trial.

**FIGURE 2:**
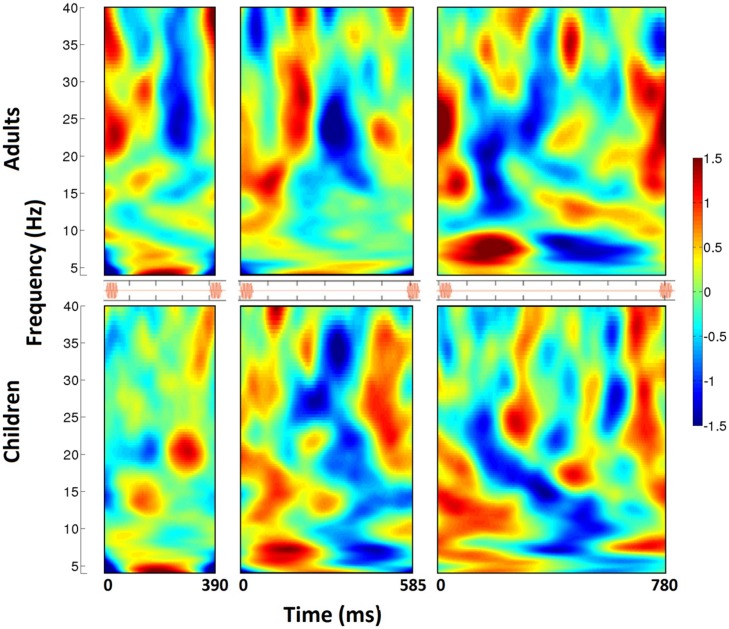
**The time-frequency spectrogram of percent power changes from right auditory cortex spatial filter for three tempo conditions (390, 585, 780 ms from left to right).** Adults are shown on the top, and children on the bottom. Analyses span from tone onset to subsequent tone onset, as visualized in the orange stimulus onset indicators for each tempo condition. From the spectrograms, a pattern of induced beta-band desychronization (negative percent change values) following each tone and a rebound (positive percent change values) before the onset of the next beat can be visualized. The timing of this pattern varies across tempo, and is stronger and more consistent in children compared to adults.

**FIGURE 3:**
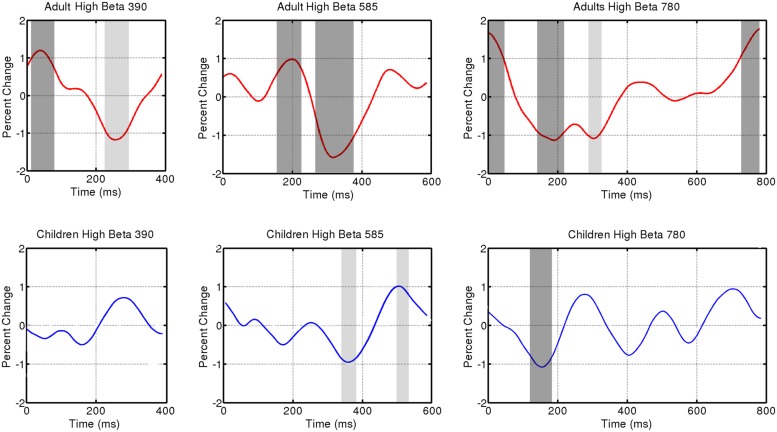
**Induced power fluctuation in high beta (20–25 Hz) activity for the three tempo conditions (390, 585, 780 ms) and two groups (adults and children).** Running one-sample *t* tests were used to determine when group fluctuations differed from zero. Light gray represents *p* < 0.05. Dark gray represents *p* < 0.01.

**FIGURE 4:**
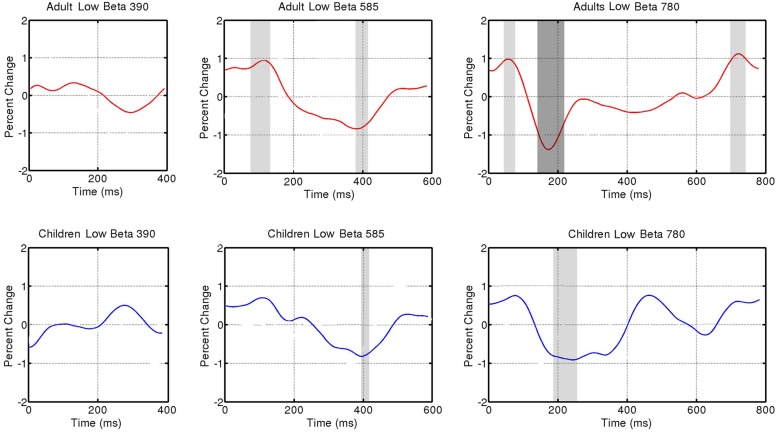
**Induced power fluctuation in low beta (15–20 Hz) activity across the three tempo conditions (390, 585, 780 ms) and two groups (adults and children).** Running one-sample *t* tests were used to determine when group fluctuations differed from zero. Light gray represents *p* < 0.05. Dark gray represents *p* < 0.01.

The individual trials, representing induced activity, were then analyzed in the frequency domain using FFTs on 200 logarithmically spaced frequencies between 2 and 50 Hz with a sliding Gaussian window ranging between 2 and 4 carrier frequency periods in length. These power spectra were averaged, and for each frequency, the calculated power was normalized to the average power across the beat window (tone onset to subsequent tone onset) and then displayed as a percent change in power over time. The spectrograms for each group (children, adults) and tempo condition were reduced to display one tone onset to the next, from 4 to 40 Hz (see **Figure 2**). For analysis, power changes in the bands 15–20 Hz (low beta) and 20–25 Hz (high beta) were averaged over frequency for each individual participant. The group averages for children and adults for each tempo condition are displayed in **Figures 3** and **4** (high beta and low beta).

## RESULTS

**Figure 3** shows fluctuations in induced power for adults and children for each tempo condition for high beta (20–25 Hz) and **Figure 4** for low beta (15–20 Hz). Adults showed a clear event-related desynchronization after tone onset in induced beta power for all three tempo conditions, followed by a rebound prior to the onset of the next tone (see **Figure 2**), similar to the previous findings reported using MEG (Fujioka et al., 2009, 2012). As with the previous MEG results, the presence of these patterns of desynchronization and rebound suggests that beta-band oscillations coming from auditory cortex reflect prediction of the timing of upcoming beats. Similarly, patterns of beta desynchronization and rebound can be seen also in the child data for the two slower tempo conditions (585, 780 ms), but there is little evidence of this pattern at the fastest tempo (390 ms). In general, the pattern of desynchronization followed by rebound in **Figures 3** and **4** appears to occur most strongly around 20–25 Hz in adults but around 15–20 Hz in children.

Finally, in adults, the tempo-dependent beta fluctuations are clearer for high than for low beta, especially for the fastest tempo (390 ms). One-sample *t* tests were used to find the time intervals during which the power change differed significantly from zero in each of the three tempo conditions. With the adult data, these *t* tests confirmed a significant dip in beta synchronization following tone onset for all three tempi in high beta and for the two slower tempi in low beta (see **Figures 3** and **4** for time ranges and significance levels). In children, one-sample *t* tests revealed a significant dip in beta synchronization following tone onset for both high and low beta for the two slower tempi (585, 780 ms), but no significant dip in beta synchronization for the fastest tempo (390 ms) for either high or low beta.

**FIGURE 5:**
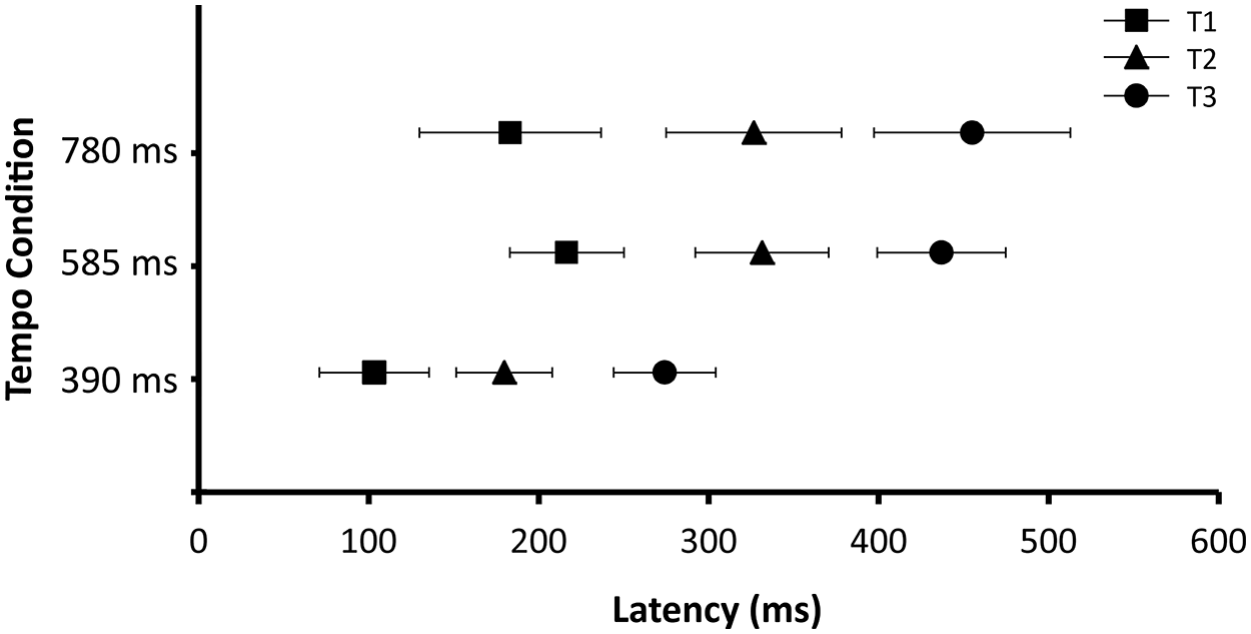
**Latency of T1 (midpoint of desynchronization), T2 (maximum point of desynchronization), and T3 (midpoint of rebound).** Data are collapsed across age group (adults and children) and beta-band (high and low). Error bars represent 95% confidence intervals.

To further investigate the timing of the beta desynchronization and rebound, for each participant the time of maximum beta desynchronization (i.e., time of minimum beta power) was determined separately for high and low beta for each tempo condition as examined in Fujioka et al. (2012). Half power was defined as half of the difference in power between the maximum synchronization and maximum desynchronization. The time courses of desynchronization and rebound were examined by identifying the time at which half power was reached prior to as well as following the point of maximum desynchronization. **Figure 5** shows the timing of these three points (midpoint of desynchronization; point of maximum desynchronization; midpoint of rebound). These three time points served as the dependent variable for three separate ANOVAs, each with tempo (390, 585, and 780 ms), group (children and adults) and frequency range (high beta, low beta) as independent variables. For each ANOVA there were no significant interactions and no significant main effects of group or frequency range of the beta-band. There were, however, main effects of tempo on the latency of the midpoint of desynchronization (*F*_(2,62)_ = 8.25, *p* < 0.001), maximum desynchronization (*F*_(2,62)_ = 18.37, *p* < 0.001) and midpoint of rebound (*F*_(2,62)_ = 21.32, *p* < 0.001). *Post hoc* analyses revealed that these time points occurred sooner for the 390 ms condition as compared to the 585 and 780 ms conditions. This result suggests that the timing of induced beta-band desynchronization and rebound does depend on the tempo, suggesting that these fluctuations are predictive in nature.

## DISCUSSION

The purpose of the present experiment was to compare oscillatory responses from auditory cortex in children and adults to isochronous beat trains with different tempi using EEG. The results reveal a pattern of induced beta-band desynchronization following each tone and a rebound before the onset of the next beat. The timing of these patterns of desynchronization and rebound varied across tempo condition. Thus induced beta-band oscillations showed predictive timing that was generally comparable to previous MEG findings (Fujioka et al., 2012). Furthermore, fluctuations in beta power appeared stronger and more consistent in adults compared to children, especially at the fastest tempo.

There were some differences between the present EEG results and previous MEG results in the details of the timing of the desynchronies. Fujioka et al. (2012), using MEG, found that the latencies of the midpoint of desynchronization and maximum desynchronization occured at consistent timepoints (∼100 and 200 ms after tone onset, respectivly) across tempo conditions, and that only the latency of the midpoint of rebound varied as a function of tempo. In the current EEG study, the latencies of all three of these timepoints varied as a function of tempo. The reasons for these differences are not clear, but might relate to differences between the ability of source filters to resolve spatially separable components in EEG vs. MEG. It is also the case that radial sources visible to EEG are invisible to MEG (Hämäläinen et al., 1993), which can lead to slight differences between the auditory responses recorded by EEG and MEG (Shahin et al., 2007). The MEG study conducted by Fujioka et al. (2012) reported patterns of tempo-dependent beta desynchronization and rebound across many brain regions, prominently including motor areas. The authors interpreted this as automatic activation of motor networks in the analysis of auditory rhythmic information. Despite the fact that we conducted our EEG analyses using source space projection to a region in auditory cortex, the EEG data still may have contained some activity generated in motor regions as well as auditory regions. Auditory and motor regions may have somewhat different time courses of beta desychrony and rebound, which may have been reflected in our data. In any case, the important result is that the pattern of fluctuation in induced beta power that follows the tempo of an isochronous auditory sequence is seen in both the present EEG results and previous MEG results.

In general, the children showed induced beta-band responses that resembled those of the adults. For example, there were no significant differences between children and adults in the latencies of the midpoint of desynchronization, maximum desynchronization, or midpoint of rebound. Across both groups there was a significant effect of tempo, suggesting that the induced beta fluctuations in both adults and children shift in phase depending on the phase of the predictive auditory events. However, the *t* tests on the amplitudes of the beta power fluctuations revealed differences in the strength and consistency of children’s and adult’s responses. In high beta, adults showed a significant desynchronization from baseline for all tempo conditions, whereas children only showed significant desynchronization for the two slower tempi. This is interesting given the fact that the range of tempi at which 8-year-old children comfortably tap (both in spontaneously tapping and in tapping entrained to an auditory beat) is not as wide as it is for adults (Drake et al., 2000a). The ability to attend to and reproduce rhythms that occur at tempi outside an ideal tempo (roughly 600 ms) develops with age (Drake et al., 2000a).

**FIGURE 6:**
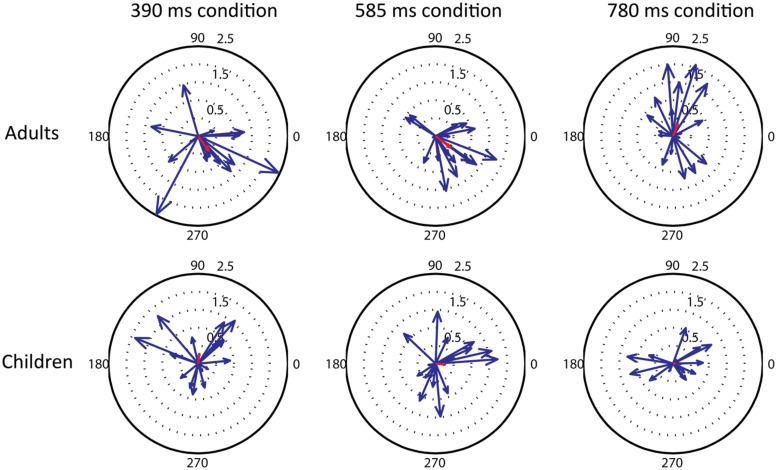
**Compass plots showing the variability between participants in the phase of the beta modulation that repeats once per beat, in the three tempo conditions (390, 585, 780 ms from right to left) and two groups (adults and children, top and bottom row).** For each individual the percent change in high beta power across a one beat interval was windowed (hamming) and an FFT calculated. The FFT component representing one beat period was converted to a vector and plotted, along with the mean vector for all subjects (shown in red).

Children showed less prominent beta desynchronizations compared to adults in the auditory cortex. Also the high-beta modulation was weaker in children than in adults. As these differences seem similar to those observed in the sensorimotor cortices associated with movement tasks between children and adults (Gaetz et al., 2010), our result might indicate a general developmental change in the beta-band oscillatory network. One possible reason for less prominent desychronizations in the group average of children compared to adults is that the time course of beta desynchronization might be more variable in children. We examined this possibility descriptively using circular plots. These plots (see **Figure 6**) show individual desynchronization data for children and adults across the three tempo conditions (390, 585, or 780 ms). Each vector represents one participant, one beat window corresponds to 360°, and the length of the vector represents the amplitude of the percent change in beta power. The direction of the vector represents the phase of the beta power change that repeats once per beat. For adults, there is some variability in phase, but most of the vectors cluster in a similar direction, indicating a similar time course of beta-band power fluctuation, reflected by the length of the average vector (in red). For children, on the other hand, there is considerable variability in phase, so that the average vector is very small. These plots suggest that neural synchronization responses to auditory rhythmic input are considerably more variable in children than in adults.

The results of the present study show that induced beta-band oscillatory responses to rhythmic sound input can be measured in children using EEG, but that such responses from auditory cortex are far from mature at 7 years of age, especially for fast tempi. Thus, the findings suggest that children’s immaturity in rhythm production discussed above might be the result of a protracted developmental trajectory for rhythm perception as well as for motoric abilities. Indeed, as children get older, their finger tapping speed becomes faster, in parallel to their motor cortical excitability assessed by TMS (Garvey et al., 2003). Especially given the variability in children’s responses, an important additional question for future research is the impact of musical training on the maturity and consistency of beta oscillations related to rhythmic processing. Finally, given that EEG has lower requirements for minimizing participant movement during recording, the finding that oscillatory responses can be measured with EEG opens the door for future studies to investigate rhythmic encoding in younger children and infants.

## CONCLUSION

This study is the first to measure induced oscillatory responses to isochronous tone sequences in children. By refining such EEG methods, it is now possible to investigate the developmental trajectory of these oscillatory responses from infancy into adulthood. Children responded in an adult-like manner at slower but not at faster tempi. This finding is consistent with the literature on spontaneous tapping rates and how the tempo range of beat perception increases with age. Future studies can focus on further disentangling the contributions of auditory perception and motoric maturity to beat perception and production.

## Conflict of Interest Statement

The authors declare that the research was conducted in the absence of any commercial or financial relationships that could be construed as a potential conflict of interest.
